# Supplementary material for: Ecology and demographic structure of an extinct ibex population in late Upper Palaeolithic Italian Alps
Source: Sci Rep. 2026 Feb 27;16:9601. doi: 10.1038/s41598-025-32389-w (PMC13009174; doi:10.1038/s41598-025-32389-w)
Supplement: Supplementary file 1 — Supplementary Material 1 [file 41598_2025_32389_MOESM1_ESM.pdf]

Supplementary Information

Ecology and demographic structure of an extinct ibex population in Late Upper Palaeolithic Italian Alps

Authors

Elena Armaroli[1*,#], Francesco Fontani[2*,#], Rocco Iacovera[2,3], Elisabetta Cilli[2], Adriana Latorre[2,4], Donata Luiselli[2], Sara Silvestrini[2], Gabriele Terlato[2], Giampaolo Dalmeri[5], Alex Fontana[5], Nicola Nannini[5], Hubert Vonhof[6], Lucio Calcagnile[7], Gianluca Quarta[7], Rossella Duches[5], Eugenio Bortolini[2], Anna Cipriani[1,8], Stefano Benazzi[2], Federico Lugli[1], Matteo Romandini[2*]

* Corresponding authors

[#] These authors have contributed equally to this work and share first authorship

Supplementary Text

Supplementary Note 1: Archaeological information

(Duches, R., Fontana, A., Nannini, N., Romandini, M., Terlato, G.)

Late Pleistocene human adaptations in the Alps

The peopling of northeastern Italy after the Last Glacial Maximum was a gradual process marked by the progressive colonisation of new territories that had been previously abandoned, following changes of vegetation and animal distribution[1,2]. Late Epigravettian penetration, firstly limited to valley floors and high plateaus around 500 m a.s.l.[3,4], reaches mid-altitude territories during the second part of the Late Glacial interstadial with the full development of a logistical occupation network at the limit between coniferous woods and alpine prairies[5]. This organisation reflects a seasonal mobility strategy that sometimes involved sites with complementary functions, located at different altitudes. Specifically, Late Epigravettian groups established semipermanent occupations on valley floors, characterized by the functional division of settlement spaces (e.g., workshops for flintknapping, dwelling structures, butchering areas, etc.) and mid-altitude seasonal camps where specialized tasks, such as hunting ungulates from the Alpine prairie (mostly *Capra ibex* and *Cervus elaphus*), meat processing, and hide and flintworking were carried out. During the Younger Dryas, climatic and environmental changes had a significant impact on Epigravettian societies[6–8], leading to several transformations in the subsistence strategies of these groups. The size and complexity of the sites suggest, in fact, the existence of higher mobility patterns compared to the previous period[9,10].

Technological analyses suggest that lithic production systems gradually simplified their structure throughout the Late Glacial interstadial, indicating a shift in technical investment from core shaping to shaping of the derived flake blank[11–13]. On the other hand, a persistence of standardized lithic backed tools used as projectile implements is attested throughout the Alleröd[11,14]. This persistence is thus a result of flexibility in retouching, framed in a progressively simplified production system. Such adaptive technology must have been encouraged by Late Glacial climatic and environmental changes and the occupation of previously inaccessible alpine territories. Thus, it can be argued that the flexibility of technical behaviours represents a key factor in the transformation of Late Epigravettian societies throughout the Late Glacial, enabling them to adapt and evolve in response to environmental, social and economic changes[11].

Riparo Dalmeri

Since its discovery in 1990, many studies have outlined a complex and exceptional picture of this rock shelter, one of the most important Palaeolithic sites in Northern Italy[15]. Among the seasonal sites in north-eastern Italy, Riparo Dalmeri is the only one that can be considered truly specialized in ibex hunting[16]. In addition to the greater abundance of *C. ibex* remains, its uniqueness lies in its characteristics of a base camp (e.g., dwelling structures, hearths, tools, ornaments and portable art), where all the family members – including children[17] – lived seasonally basing much of their subsistence on ibex[18,19]. Paleoenvironmental reconstruction indicates an open alpine prairie with some emerging wooded areas of pines and larches nearby[5,20]. The presence of species other than ibex in the faunal record, including other ungulates, small mammals, carnivores, birds and fishes indicates that the territory exploited by hunter-gatherers was quite large, extending from alpine meadows to the valley floor of the Brenta River[21,22]. However, ibex was the most hunted species and the main reason for site occupation. The great importance this animal had to Epigravettian hunter-gatherers is also stressed by its representation on some painted stones and the presence of pits intentionally filled with ibex cranial bones and horns. These findings seem to be related to a complex ritual that could broaden the interpretation of the site to the symbolic and ritual sphere, at least in the first phase of occupation[15,19,23].

The site, located at about 1240 m a.s.l., overlooks the head of a small periglacial valley, a tributary of the deep Valsugana canyon (Trentino) crossed by the Brenta River. The morphogenesis of the rock shelter results from the differential erosion of the stratified limestone bedrock (oolitic lithofacies of the Jurassic Rotzo Formation), caused by the combined effects of carbonate dissolution and cryogenic processes during the Last Glacial Maximum[20,23]. The shelter faces N-E and extends NNW-SSE for 30 m. The rock overhang extends up to 7 m, with an actual height of 4 m above the ground. The shelter's stratigraphic sequence spans from the end of the Upper Pleistocene to the Holocene and it is approximately 4.5 m thick. Inside the rock shelter, the deposit has been divided from bottom to top into the following stratigraphic complexes[15,23,24]:

- The stratigraphic sequence preceding the settlement phase consists of a sequence of thermoclastic breccias, indicative of generally cold and humid conditions. Specifically, an early deposit associated with carbonate silts of karst and/or colluvial origin was identified directly on top of the bedrock. Upward the breccia is enriched with a matrix of aeolic origin. Finally, an open-work breccia with clasts characterized by thin capping, typical of climatic conditions involving frost. Stratigraphic unit (SU) 50 is distinguished by its enrichment in a clay matrix of karst and/or colluvial origin.
- Phase 1. The first phase of human occupation of the shelter involves the raising of a structure near the dripline of the rock shelter. It is represented by an accumulation of collapse blocks (SU 74), at the top of which was found a large stone with an

anthropomorphic figure (RD 211). This structure partially covers a breccia with an organic matrix (SU 65), which has a strong anthropogenic component (i.e., lithic industry, faunal remains, and charcoals) associated with numerous ochre-painted stones (n = 267). SU 65 constitutes a patch that tapers towards the inner area of the shelter, reaching a maximum thickness of 45 cm near the present dripline. Most of the painted stones are found within this unit, while some are found scattered in the innermost part of the shelter, on top of a cryoclastic breccia (SU 15a). The stones' distribution forms a band of about 30 m^2, 4 m wide, east-west oriented and oblique to the alignment of the innermost wall. Human occupation during this phase is further supported by the presence of two hearths in the eastern part of the shelter and the remains of a dwelling structure with a 4 m diameter.

- Phase 2. The later Epigravettian dwelling floors (SU 26c, 26b/14b) are characterized by the superposition of Ah-type organic horizons related to anthropogenic activity. These horizons developed on a parent material of cryoclastic breccia (SU 15a), enriched with a silty, micaceous, dark, highly organic matrix containing abundant anthropogenic remains. The two main archaeological horizons (SU 14-26b and SU 26c), found in stratigraphic succession, were excavated over an area of 84 m^2 and yielded a substantial assemblage of lithic and bone artefacts, animal remains, human deciduous teeth, ornamental objects, and engraved stone tool cortex. The identified features allowed the settlement area to be divided into a western sector, where a sub-circular hut was still in use, and an eastern sector, where hearths were located. Inside the hut, concentrations of faunal remains and an area showing traces of burning were identified.
- Phase 3. The upper sequence of the shelter is composed of stratified breccias subdivided into several layers (SU 6, 7, 9, 21, 26, 24). These deposits are mainly supported by a silty matrix, although some areas exhibit complete or partial clastic support. The matrix is largely formed of biogenic "moonmilk", whose deposition is associated with the onset of temperate climatic conditions marked by higher precipitation and temperatures, typical of the Holocene Climatic Optimum. Attribution of these layers to the Holocene is not confirmed by radiocarbon dating. However, a preliminary tecno-typological analysis indicates that the lithic assemblage corresponds to a Late Epigravettian industry, with distinctive traits related to the final stages of this chrono-cultural complex[10,14,25]. In particular, the presence of geometric microlithic armatures and trapezoidal bitruncations suggests that some layers (SU 21 and 26) may date to the Younger Dryas or the Pleistocene-Holocene transition.

The external succession of Riparo Dalmeri is divided into several units. Some correspond laterally (heterotopically) to those in the inner part of the shelter, while others are of more recent age[26]. Above the occupation layers dated to the Late Glacial interstadial, the external sequence shows a stratigraphic gap roughly corresponding to the Younger Dryas. Sedimentation resumed at the Pleistocene-Holocene transition, culminating in a younger phase of input around the Preboreal-Boreal shift[26]. The lower part of the external succession is composed of coarse clastic sediments with substantial anthropogenic input (SU 83, 84, 85, 85a, 88, 89, and 92, structure 86). These deposits laterally continue the habitation surfaces of the inner stratigraphic sequence (Phases 1 and 2). Above them lie units 64, 77 (formerly 64a), 78 (formerly 64b), 79, and 81. The outermost part of the sequence consists, from bottom to top, of units 85, 82, 76, 75, 91 and 90 (see Supplementary Fig. S1).

The group of units 64, 77, 78, 79, and 81 forms a sigmoidal, lenticular deposit with lateral interfaces dipping 45° inward and outward from the shelter; its upper interface is nearly horizontal, while the lower interface slopes outward at 10°–15°. The group of units 90, 91, 75, and 76 forms a biconcave sequence, whereas the basal complex (units 82, 83, and 85) consists of tabular layers slightly inclined toward the exterior[26].

Regarding the younger sedimentary phase of the external succession (units 64, 77, 78, 79, and 81), discrepancies emerge between radiocarbon dating and chrono-cultural attribution based on the lithic industry. Previous radiocarbon dates obtained from charcoal[26] pointed to a younger phase of human occupation during the Preboreal-Boreal shift. However, the lithic assemblage does not correspond to the First or Early Mesolithic[27,28]. Since no evidence of Mesolithic frequentation has been documented, these layers may instead result from reworking of Late Epigravettian anthropogenic material from the inner part of the shelter. Consequently, the Holocene charcoal dates may reflect paleosol formation rather than the primary deposition of anthropogenic material. The new radiocarbon dates presented in this study help clarify these discrepancies.

Supplementary Figure S1. Stratigraphic section (top) and planimetry of Riparo Dalmeri. Drawing by Michele Bassetti, Giampaolo Dalmeri, Stefano Neri, and Diego E. Angelucci; graphical editing by Anna Bernardo and Diego E. Angelucci for Angelucci et al.[26]. Image courtesy of Giampaolo Dalmeri.

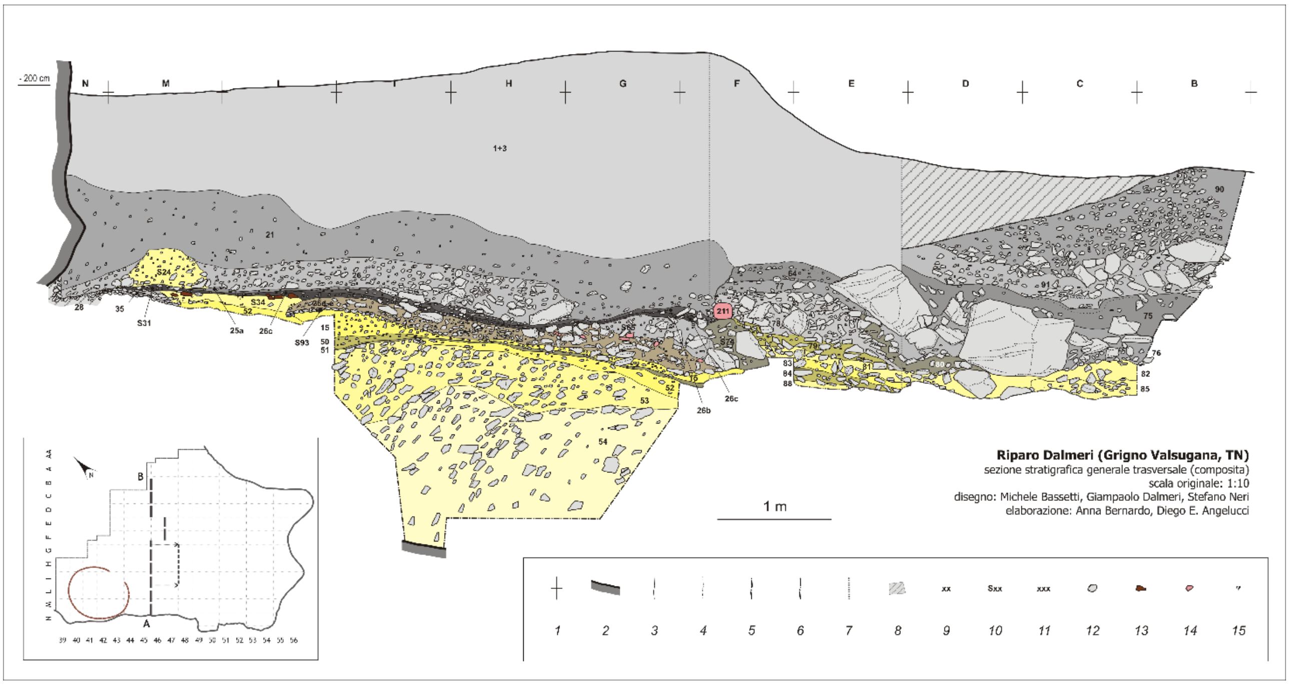

Riparo Cogola

The rock shelter site of Riparo Cogola is located 1070 m a.s.l. on the northern edge of the Sette Comuni Plateau, on the Vicentine Alps (Trento, NE Italy). Excavations conducted between 1999 and 2002 by researchers from the Museo Tridentino di Scienze Naturali identified three main periods of frequentation: the most ancient during the Final Epigravettian (SU 19, radiocarbon dated between 12600-11950; 12900-12450 cal. BP); a transitional phase between the Epigravettian and the Sauveterrian (SU 18, radiocarbon dated at 11250-11150 cal. BP); and the Sauveterrian (SU 17-16, the latter radiocarbon dated 10750-10550 cal. BP)[20]. In all three phases, the primary hunting prey consisted of ungulates (red deer, roe deer, ibex, and chamois), while wild boar was present only in the first two phases and elk only in the Early Sauveterrian. The analysis of the bones remains indicates that ibex carcasses were taken into the shelter whole and then butchered, as evidenced by the large number of skeletal elements with cut marks and percussion marks. In contrast, a selective butchering process was applied to deer carcasses, with only the anterior and posterior portions being brought inside the shelter. Studies on the age profiles of individuals, combined with thin sections of teeth to determine the age at death of ibex and chamois, show that most of the animals were killed during the summer–autumn period, suggesting that human hunter-gatherer groups occupied the rock shelter predominantly at this time of year[29]. The two samples selected for paleogenetic analysis were directly radiocarbon dated following the same procedure described in

the Materials and Methods section. Results align with the anthropic frequentation phases of the rock shelter, with sample 0830 radiocarbon date 12650-12500 cal. BP (1σ) and sample 0831 radiocarbon dated 11150-10800 cal. BP (1σ).

Romagnano Loc III

The rock shelter site of Romagnano Loc III was discovered in 1968 during quarrying activities. Located at 210 m a.s.l., approximately 10 km south of Trento (NE Italy), the site hosts an extremely rich and well-preserved archaeological deposit, spanning from the Mesolithic to the Iron Age[28,30–34]. The Mesolithic deposits were excavated between 1971 and 1973 by researchers from the Museo Tridentino di Scienze Naturali and the Università di Ferrara[35]. Several layers were identified and systematically radiocarbon dated from 11703 to 7280 cal. BP[28,36]. The faunal assemblage collected from the Middle and Late Sauveterrian levels AC primarily consists of ungulates, with a significant prevalence of *Capra ibex* and *Cervus elaphus*. The two *C. ibex* samples analysed in this paper (ID 6123 and 6124) were found in layer AC8. This stratigraphic unit was dated to 10500-10250 cal. BP and was associated with Middle Sauveterrian lithic technology.

Supplementary Note 2: Archaeozoological data

(Duches, R., Fontana, A., Nannini, N., Romandini, M., Terlato, G.)

Archaeozoological studies conducted on the faunistic assemblage from Riparo Dalmeri[16,19,21,37–42] have provided valuable insights into the site's economy and clarified the methods of animal exploitation. These analyses considered more than 120,000 bones, among which over 15,000 specimens were determined to species or genus level. Since earlier studies did not group the material according to the phases of human occupation, we present in Supplementary Data 1 a subset of almost 6,500 specimens that we were able to confidently assign to the three phases discussed in this study. The list of remains specifically dated for the purpose of this study is presented in Supplementary Data 2. In all phases, hunting was primarily focused on ibex (*Capra ibex*), with a lesser emphasis on red deer (*Cervus elaphus*). Wild boar (*Sus scrofa*), roe deer (*Capreolus capreolus*), elk (*Alces alces*), and chamois (*Rupicapra rupicapra*) are represented by very few remains. Among carnivores, the most numerous remains belong to the bear (*Ursus arctos*), followed by fewer elements attributed to the fox (*Vulpes vulpes*), wolf (*Canis lupus*), and badger (*Meles meles*). Remains of the hare (*Lepus* sp.), beaver (*Castor fiber*), marmot (*Marmota marmota*), and European hedgehog (*Erinaceus europaeus*) are exceptionally rare. Fish remains were also identified, with Cyprinids comprising 90% of the total. The majority of these remains belong to the barbel (*Barbus plebejus*) and the chub (*Leuciscus cephalus*), while fewer are attributed to trout (*Salmo trutta*) and grayling (*Thymallus thymallus*). Pike (*Esox lucius*) remains are extremely rare. The presence of chub and trout individuals measuring around 30/40 cm supports the hypothesis that a selective fishing strategy (through hooks, harpoons or bow and arrows projectile systems) was the primary method of capture[43]. The bird remains (n = 146) are mainly attributable to Galliformes, including Tetraoninae, such as the black grouse (*Tetrao tetrix*), Lagopedes (rock ptarmigan, *Lagopus* cfr. *mutus*, and willow ptarmigan, *Lagopus* cfr. *lagopus*) and small Phasianidae (common quail, *Coturnix coturnix*). Passeriformes are also relatively abundant. Most of these birds were undoubtedly introduced into the shelter by humans, as indicated by anthropogenic traces, particularly on the Galliformes remains[37].

Hunting of ungulates was primarily focused on early-stage adults and older adults, likely to maximize meat yield. The seasonality of human frequentation, determined by the presence of ungulate teeth from very young and young individuals, and confirmed through the analysis of tooth thin sections, indicates that hunting occurred between summer and autumn[19,39,40,42,44]. Butchering marks are abundant both on ibex and red deer bones. Archaeozoological data, based on the comparative frequencies of recovered skeletal remains, reveal a differential transport of animal carcasses from the initial kill site to the rock shelter. Hunters typically brought entire carcasses of medium-sized prey to the shelter, while only specific portions of larger animals, such as red deer, were selected for

transport. Consistently, the frequency of ibex skeletal portions indicates that all elements of the carcass are represented, with a balanced distribution between the right and the left halves. The scarcity of some elements is attributed to differential bone preservation and the high rate of fragmentation, which in turn results from intense anthropogenic exploitation of the carcass. The spatial distribution of animal remains in the 26c anthropogenic horizon revealed a concentration of bones exhibiting cut marks and percussion marks. This area was therefore interpreted as dedicated to the processing and butchering of ibex carcasses. The fracturing of bones took place inside the shelter, as evidenced by the higher frequency of percussion cones compared to the number of percussion marks found on the diaphysis. This data also suggests that the shelter was systematically cleared of larger fragments[40]. Additionally, the concentration of burnt remains points to areas where fires were frequently lit, with bones also used as fuel. Occasional gnawing marks, together with a few bones attributed to bear and wolf cubs, indicate the alternate occupation of the shelter by carnivores and humans.
The results of the metric analysis on third molars (M3) from Riparo Dalmeri are summarized in Supplementary Table S1 and compared with data published in[19] (Supplementary Table S2).

Supplementary Table S1. Max anterior-posterior diameter (MAP) and transversal diameter (TD) measurements (in mm) for M3 teeth in the Dalmeri specimens. Age classes: JAd (young adults), Ad I (early-stage adults), and Ad II (older adults).

ID	*TD*	*MAP*	*sex*	*Age*
RD_8153	10,3	27,8	NA	Ad II
RD_8155	10	27,2	NA	Ad II
RD_8157	9,8	27	NA	JAd
RD_8158	9,7	28,2	Female	Ad I
RD_8161	9	26,1	Female	Ad II
RD_8164	8,48	27,4	NA	JAd
RD_8165	9,32	29,8	NA	Ad I
RD_8166	9,09	28,8	NA	Ad I
RD_8170	9,6	29,2	NA	JAd
RD_8171	9,6	27	NA	Ad I
RD_8172	8,9	24,4	NA	Ad I
RD_8175	10,2	28,1	Female	JAd
RD_8179	10	28	Male	Ad II
RD_8180	10,1	28,3	NA	Ad I
RD_8181	10,1	29,2	NA	Ad I
RD_8182	10,2	27,9	NA	JAd

RD_8183	10,3	30,9	NA	Ad I
RD_8184	10,2	27,7	Male	Ad I
RD_8185	10	29,4	Male	Ad I
RD_8187	10,6	29	NA	Ad I
RD_8200	9	27,6	NA	Ad II
RD_8201	10,3	29,5	NA	JAd
RD_8202	10,6	27,6	Male	Ad II
RD_8203	10,1	26,2	Male	Ad II
RD_8204	10	28,7	Female	Ad II

Supplementary Table S2. Comparison of MAP measures of lower M3 registered in this study and in several sites reported in[19]. All sites are listed chronologically.

Site	*n. samples*	*Max*	*Min*
Grotta del Broion	30	32,2	23,4
Grotta di Fumane	7	30,7	28,1
Paglicci Str.21-28	54	33,6	27,4
Cala Str.Q	2	28,1	27,4
Paglicci Str.6-1	6	30	24
Tagliente	6	32,4	26
Dalmeri (this study)	25	30,9	24,4
Dalmeri (Fiore and Tagliacozzo, 2006)	14	30,59	26,29
Romagnano III	6	27,3	23
Cogne (modern populationi)	7	24,5	23

The graph in Supplementary Fig. S2 shows that the median MAP value for Dalmeri is 28 mm, which falls within the total measurement range observed at other sites, spanning from 23 mm to 33,6 mm. This range highlights a notable degree of variability in M3 dimensions. The comparison suggests differences in dental size between older and more recent populations. Specifically, the M3 dimensions from Dalmeri are comparable to those from Grotta del Broion[16] — a site in northeastern Italy with a rich Middle to Late Upper Palaeolithic deposit — but exhibit variations when compared to more recent populations, particularly those from Romagnano III and modern ibex specimens. The results suggest some variation in M3 dimensions based on both ontogenetic age and sex. However, no significant correlation is observed between the measurements, age, and sex of the individuals

from Dalmeri. Adult males generally exhibit larger M3 dimensions than females, though some overlap is present (Supplementary Fig. S3).

Supplementary Figure S2. MAP median values of lower M3 teeth from Dalmeri, compared with other sites spanning from the Middle Palaeolithic to the early Holocene, as well as some modern ibex specimens. Data from other sites are from[19].

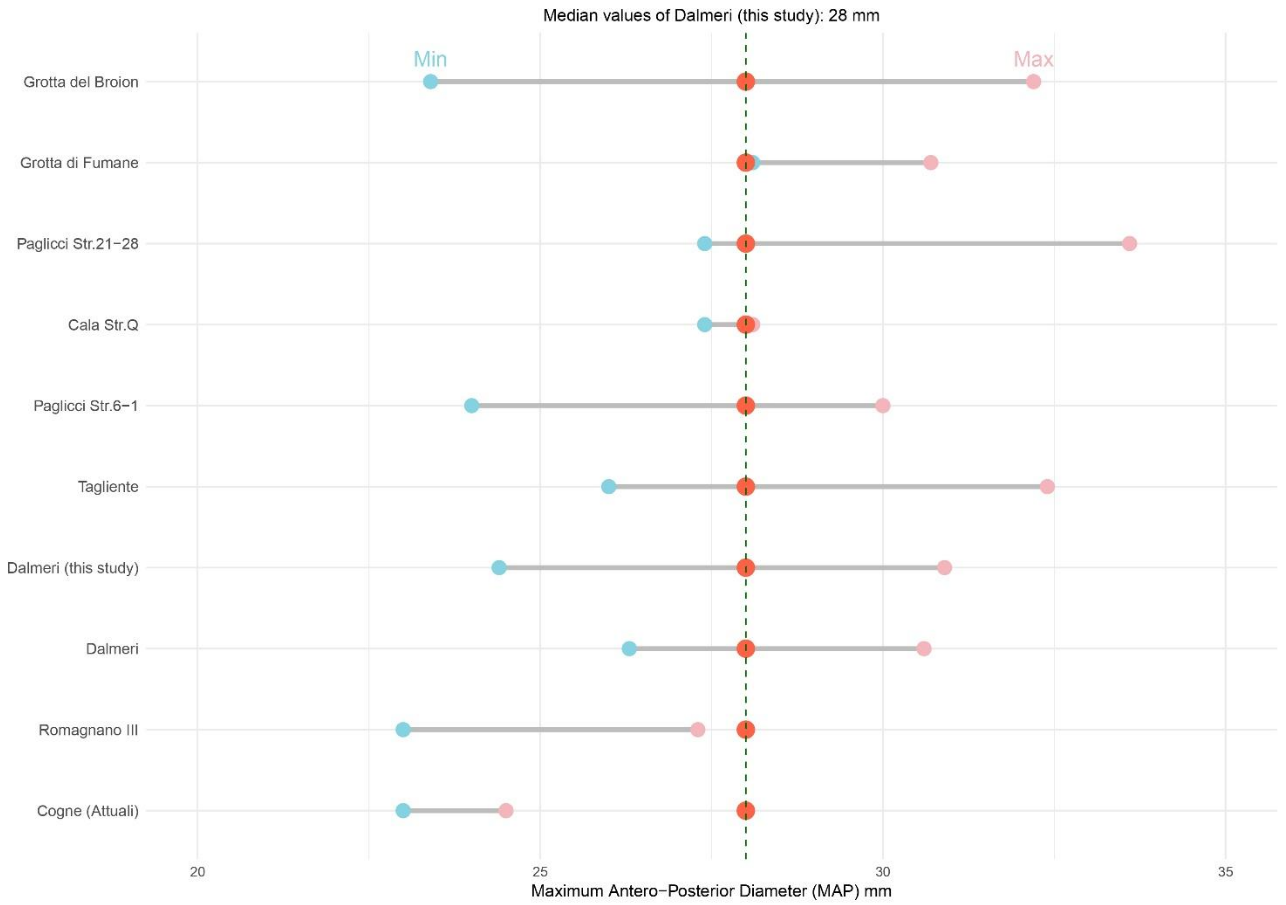

Supplementary Figure S3. Scatter plot of M3 measurements from Dalmeri, analyzing the relationship between age and sex of individuals using Kendall's tau correlation. Maximum Antero-Posterior Diameter (MAP) and transversal diameter (TD) in millimetres. Age classes: JAd (young adults), Ad I (early-stage adults), and Ad II (older adults).

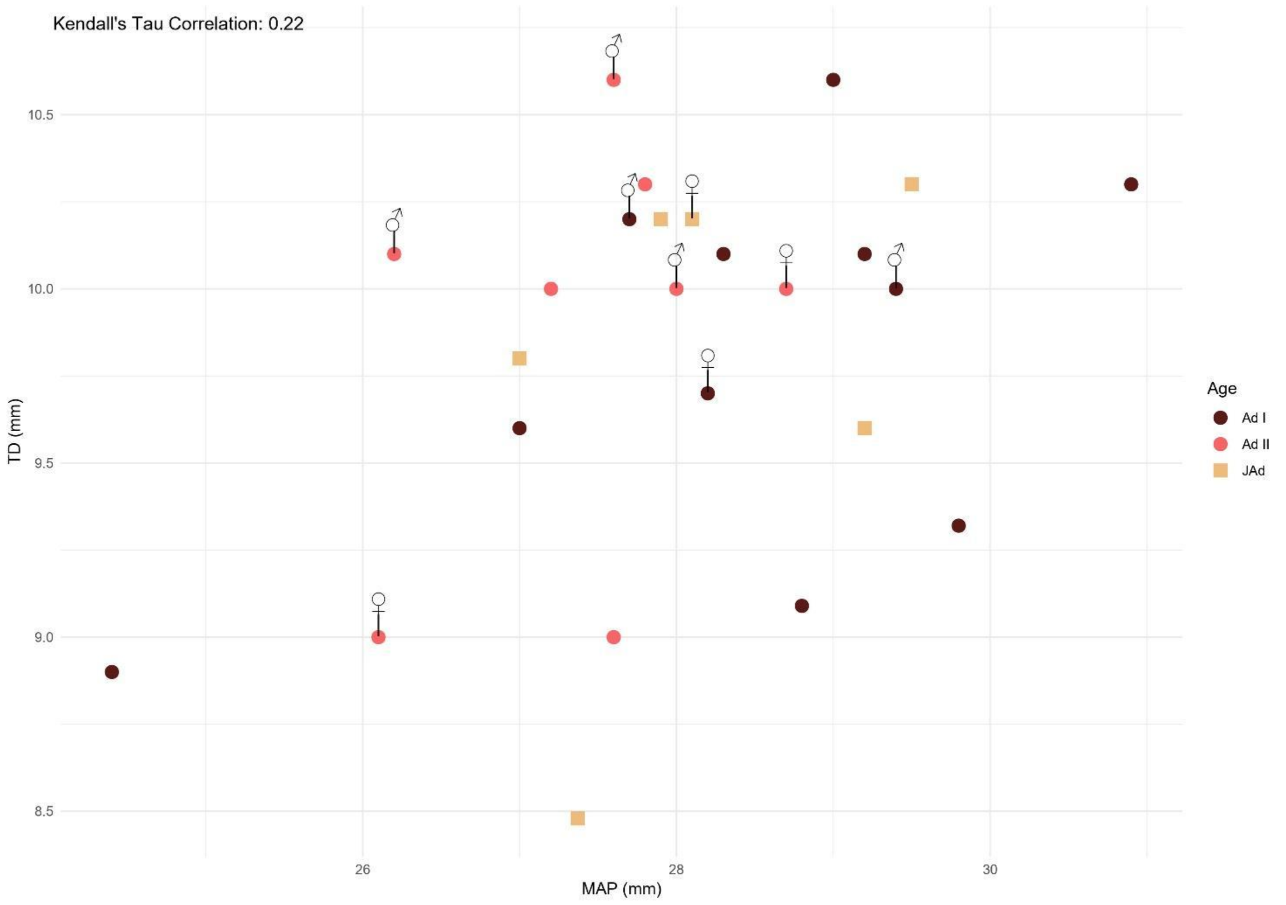

Supplementary Note 3: Paleoproteomics

(Armaroli, E., Silvestrini, S., Lugli, F.)

We collated a protein reference database containing entries for COL1A1, COL1A2, COL2A1, CO4A1, AMELX, AMELY, TUFT1, MMP20, KLK4, AMBN, ALB and ENAM of *Bos taurus*, *Sus scrofa*, *Ovis aries*, *Capra hircus*, *Capra ibex* and *Homo sapiens*, derived from UniProt. Proteomic data analysis was performed through MaxQuant (MQ) v2.1.0.0 using unspecific digestion and setting deamidation (NQ), phosphorylation (ST), and oxidation (M) as variable PTMs. Peptides' length was allowed to be between 7 and 25 amino acids, with a minimum score of 35, and filtered to FDR = 1% at peptide and protein level; other settings were left as default. Preliminary Mascot searches (vs. SwissProt) on modern *Capra ibex* specimens with known sex, indicated that UniProt reviewed amelogenin sequences of *Bos taurus* show the expected pattern among sexes (i.e., AMELY in male only; Supplementary Fig. S4), while unreviewed and incomplete AMEL sequences of *Capra ibex*, *Ovis aries* and *Capra hircus* show worse coverage and no sex-related differences. We therefore decided to rely on AMEL entries for *Bos taurus* to estimate the sex of fossil samples. All individuals with at least one specific-AMELY peptide after MQ search were estimated as males, after validating this approach on the three modern samples (note e.g. that ibex_mod_M1 shows a single AMELY peptide and is a male); all the peptides were then pooled and their intensities summed (Supplementary Fig. S5).

Supplementary Figure S4. Mascot fragmentation spectra of sexual-dimorphic AMELY (Q99004) and AMELX_BOVIN (P02817) peptides, obtained from modern *C. ibex* samples. No AMELY peptides were found in the modern female individual. The main sexual-dimorphic region observed is highlighted in yellow; sequences of AMELX_BOVIN and AMELY_BOVIN were aligned with Clustal O (1.2.4).

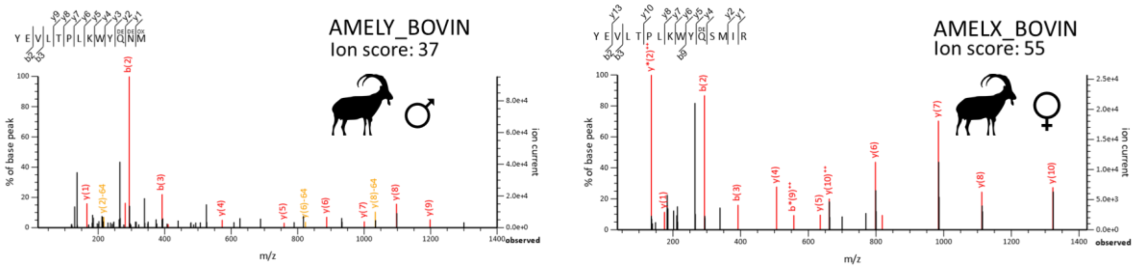


```
sp|P02817|AMELX_BOVIN MGTWILFACLLGAAFSMPLPPHPGHPGYINFSYEVLTPLKWYQSMIRHPYPSYGYEPMGG 60
sp|Q99004|AMELY_BOVIN MGTWILFACLLGAAYSMPLPPHPGHPGYINFSYEVLTPLKWYQNMLRYPYPSYGYEPVGG 60
                      **************:****************************.*:*:*********:**
```

Supplementary Figure S5. Total intensities of razor-AMELX and AMELY peptides from fossil and modern ('ibex_mod') *C. ibex* enamel samples after MaxQuant search. Half-circles have an AMELY intensity equal to 0.

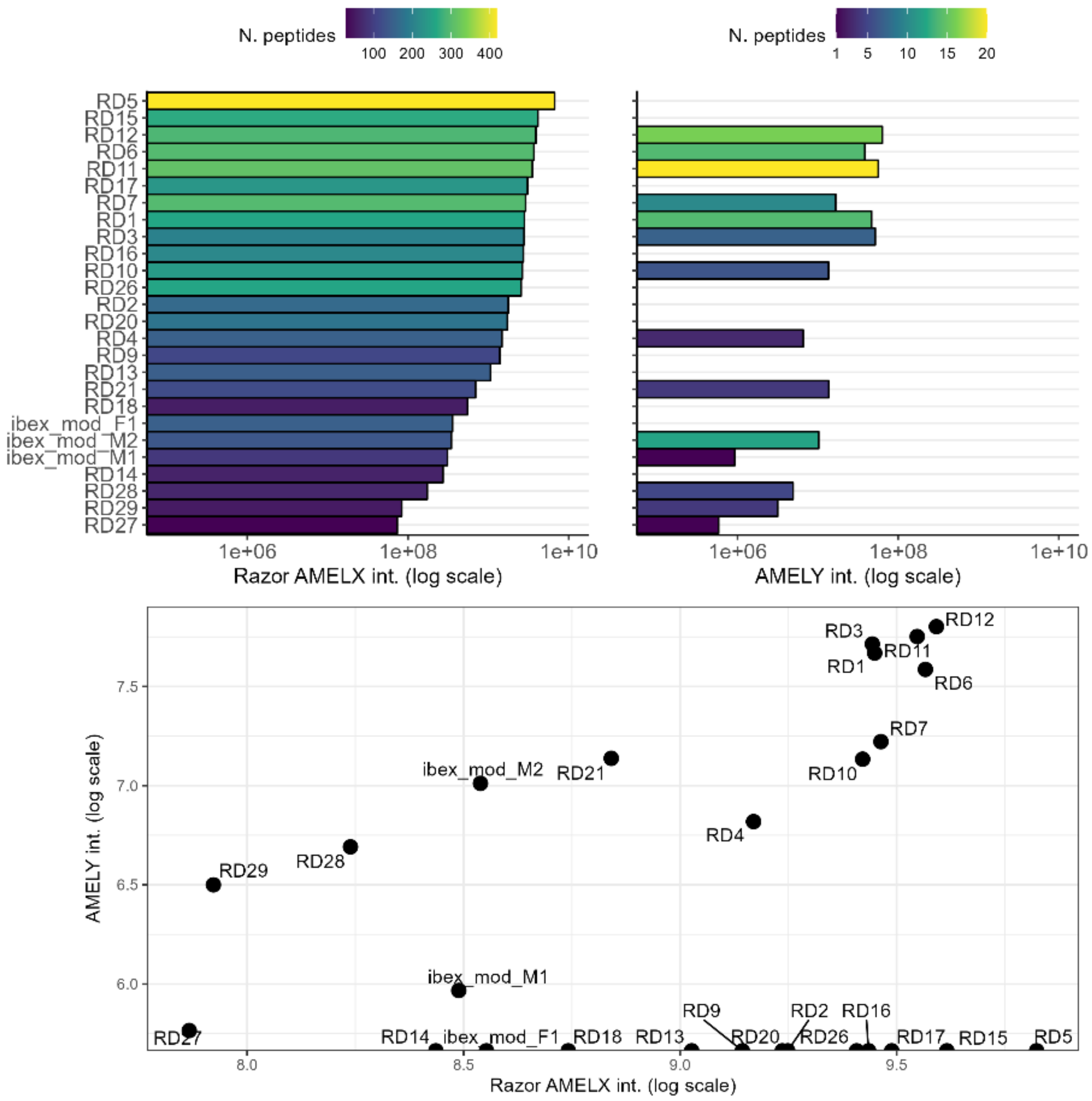

An initial comparison with DNA sexing indicated proteomic sex estimation of RD9 as female false positive, a known issue in amelogenin-based sex estimation; all the other individuals agreed with DNA. We thus decided to perform a second search run using Mascot for all those samples that resulted as females through MQ; the two search engines are indeed known to occasionally provide different matching peptides (e.g.[47]). For the Mascot search, we used the same PTMs, FDR threshold and reference fasta dataset; data were additionally searched against cRAP (contaminants dataset); only peptides with an ion score > 20 were considered as identified. After this second search, additional AMELY peptides were found in three individuals previously estimated as females, namely

RD9, RD14, and RD16. All the proteomic sexes agree with DNA estimation (Supplementary Table S3).

Supplementary Table S3. IDs correspondence and AMELY presence (●)/absence (empty cell) after MaxQuant (MQ) and Mascot searches; females only (after MQ search) were searched with Mascot; final proteomic sex estimations were performed as a combination of the two search results.

Sample ID	*Proteomic ID*	*DNA sex*	*AMELY_BOVIN MQ search*	*AMELY_BOVIN Mascot search*	*Final proteomic sex estimation*
RD_8156	RD18	n.d.			F
RD_8158	RD17	F			F
RD_8159	RD27	M	●	n.d.	M
RD_8161	RD13	F			F
RD_8163	RD15	F			F
RD_8168	RD14	n.d.		●	M
RD_8173	RD11	n.d.	●	n.d.	M
RD_8175	RD16	n.d.		●	M
RD_8177	RD26	n.d.			F
RD_8179	RD9	M		●	M
RD_8184	RD10	n.d.	●	n.d.	M
RD_8185	RD12	n.d.	●	n.d.	M
RD_8189	RD3	n.d.	●	n.d.	M
RD_8190	RD20	F			F
RD_8191	RD1	n.d.	●	n.d.	M
RD_8192	RD28	M	●	n.d.	M
RD_8193	RD2	n.d.			F
RD_8194	RD29	M	●	n.d.	M
RD_8195	RD4	n.d.	●	n.d.	M
RD_8198	RD21	n.d.	●	n.d.	M
RD_8202	RD7	M	●	n.d.	M
RD_8203	RD6	M	●	n.d.	M
RD_8204	RD5	F			F

Deamidation levels were calculated following the approach of[48], but developing an R script ad-hoc (R version 4.0.5, "Shake and Throw", doi: 10.5281/zenodo.15024003). In brief, the number of deamidated N and Q were counted from the ‘evidence.txt’ file (MaxQuant output) for all the peptide-to-spectrum matches (PSM) and normalized to the number of unmodified N-Q. These values were grouped by peptides per charge state and a weighted average was calculated based on the intensity of each PSM. Values were then averaged by peptide. The average deamidation rate and confidence interval per sample were bootstrapped (n = 1000), using the previously-obtained peptide average values. Overall, both modern and fossil samples show highly deamidated Q residues (median > 0.90; with 1 = fully deamidated, 0 = non-deamidated) and variable deamidation rates of N residues (Supplementary Fig. S6). Notably, a modern sample (ibex_mod_M1) shows the lowest median N deamidation rate of the dataset (0.62); yet, the other modern samples (ibex_mod_M2 and

ibex_mod_F1) show N deamidation rates akin to fossil samples. This evidence agrees with previous works, where the deamidation rate of modern samples is highly-variable and eventually showing high rates[49]. Peptides' lengths of AMBN, AMEL, and ENAM have been estimated for fossil and modern samples, with the latter showing a higher modal peptide length (14 AAs; Supplementary Fig. S7) as expected. In addition, compared to modern, fossil samples show a higher number of short peptide chains (<14 AAs), possibly due to diagenetic hydrolysis. All the raw data (including a blank), the MaxQuant evidence file, and the deamidation script were uploaded to Zenodo (https://doi.org/10.5281/zenodo.15024003).

Supplementary Figure S6. Asparagine (N) and glutamine (Q) deamination rates for modern (*mod*) and fossil enamel specimens of *C. ibex*, as calculated from the 'evidence.txt' MaxQuant output; facets are ordered based on the median N deamidation rate. Boxplots represent the bootstrapped dataset after generating n = 1000 (re)samples with replacement for each individual. The number of peptides used for calculations is given next to the sample name.

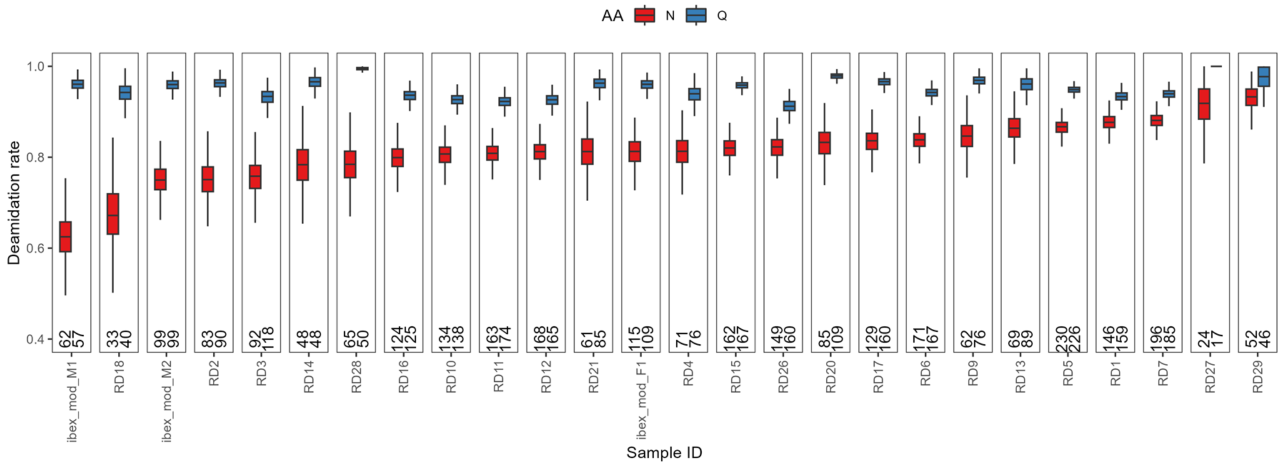

Supplementary Figure S7. Lengths (number of AAs) of AMBN, AMEL, and ENAM peptides for fossil and modern samples of *C. ibex*. Facets are ordered based on the modal peptide length of each sample. Red triangles represent statistical modes.

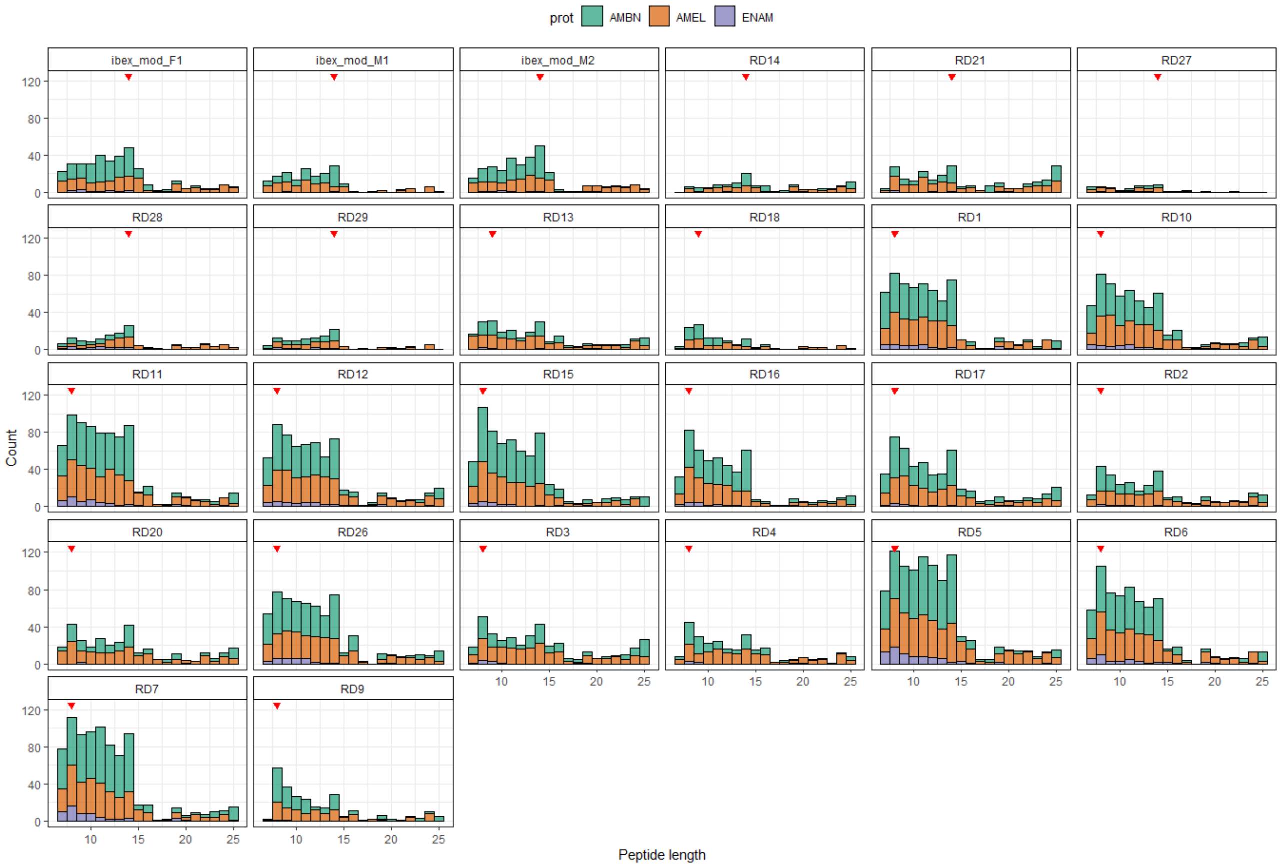

Supplementary Note 4: Phylogenetic analysis of ancient mitogenomes

(Fontani, F., Iacovera, R., Luiselli D., Cilli, E.)

For phylogenetic analysis of high quality mitogenomes (>10x), we reconstructed three different datasets. All of the newly generated data from Riparo Dalmeri displayed distribution of C-to-T transition patterns in the first position at 5' ends ranging from 30% to 47%, and the mean length of most mapped fragments spanned from 52 to 76 bp, indicating that the ancient DNA was authentic. The dataset "Capra_all" contained 48 *Capra ibex* samples (29 modern, 7 historic, 6 ancient, and 6 samples from Riparo Dalmeri), 4 *Capra pyrenaica*, 6 *Capra aegagrus*, 2 *Capra sibirica*, 1 *Capra falconer*i, 2 *Capra nubiana*, 16 *Capra hircus,* and 5 *Ovis aries*. For this dataset, we have removed the entire D-loop due to its high intraspecific variation in the control region, for a total of 15,529 bp. The dataset "Capra_ibex/pyrenaica" contained 44 *Capra ibex* (29 recent, 7 historic, 6 ancient and the 6 samples from Riparo Dalmeri) and 4 *Capra pyrenaica*. This dataset contained 15,603 sites of the 16,716 possible sites. We furthermore produced a third dataset "Capra ibex" composed of entire mitochondria, which only contained *Capra ibex* specimens. For each dataset, we used ClustalW/X[45] to align the sequences. All the historical and ancient mitochondrial sequences used to construct the three datasets have a mean coverage of ≥ 9x and yielded a robust phylogenetic tree (Fig. 9). In fact, mitogenomes characterized by a coverage < 9x occupy unstable positions in the topology of the "low coverage" maximum likelihood tree (Supplementary Fig. S8) computed from the dataset "Capra_all_low" (Supplementary Data 4). This instability not only results in questionable phylogenetic placements for these samples but also negatively impacts the statistical robustness of the associated nodes, as evidenced by significantly reduced bootstrap values. Including such low-coverage samples compromises the overall resolution of the tree, increasing uncertainty in the definition of phylogenetic relationships and invalidating the reliability of the analysis. Therefore, their exclusion is necessary to preserve the quality and consistency of the phylogenetic inference and subsequent analyses. Based on these criteria, five mitochondrial sequences from Riparo Dalmeri (RD_8163, RD_8190, RD_8202, RD_8203 and RD_8204), the two samples from Loc di Romagnano (6123, 6124), the two samples from Riparo Cogola (830, 831) and three mitochondrial sequences from Robin et al. 2022 (Gro1, Pil2 and Zue2) were discarded from the analysis.

Supplementary Figure S8. Maximum likelihood tree with low coverage samples.

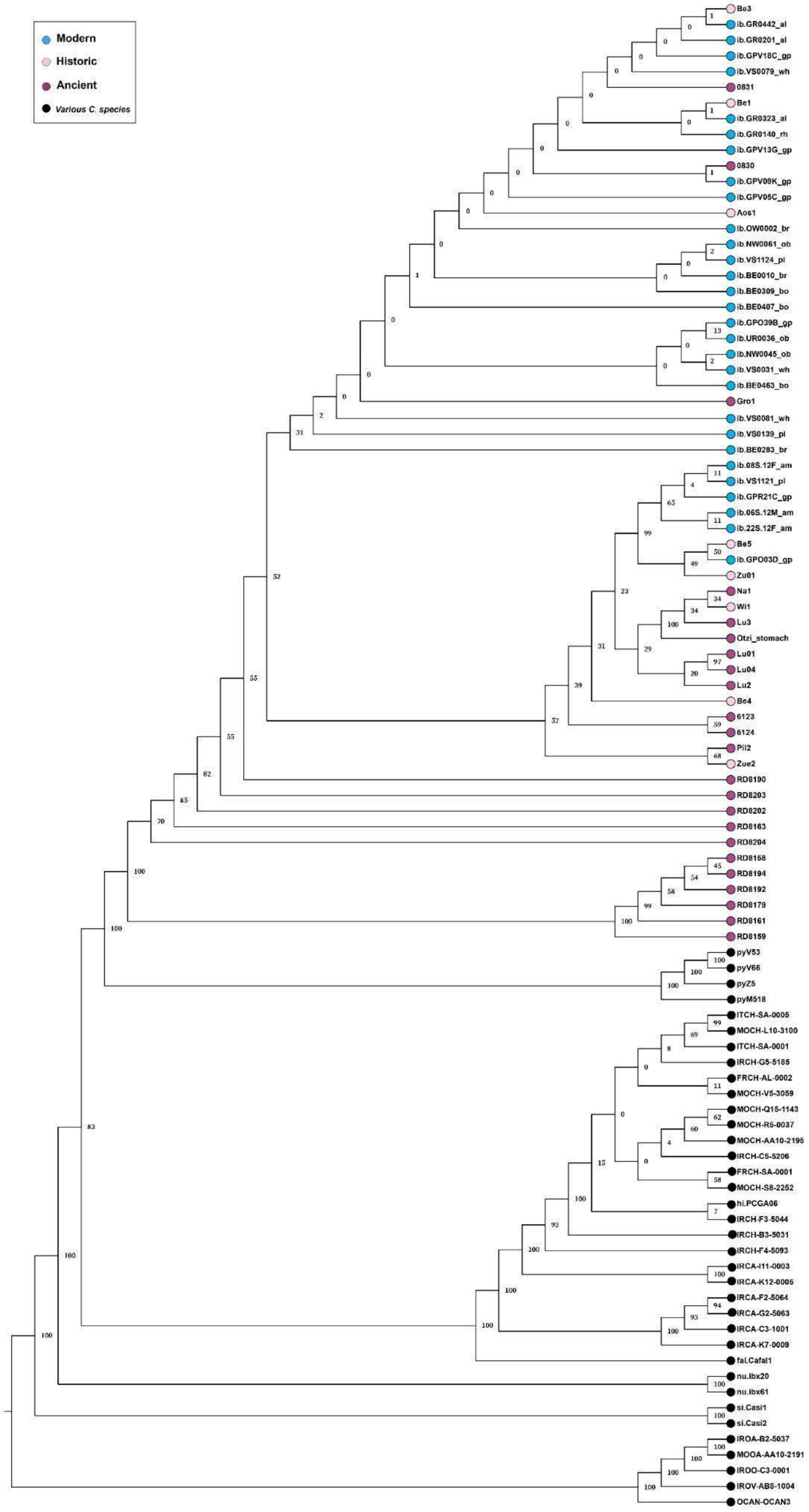

Interestingly, all the haplotypes reconstructed from the Dalmeri mitogenomes (haplogroup A) are unique, and no genetic affinity is visible between samples from the same archaeological phase, suggesting that the population remained relatively large across different phases despite intensive hunting. Haplogroups B and C, also made up only of unique haplotypes, consist of the remaining ancient samples, along with the outlier Wi1. The two remaining haplogroups, D and E, consist exclusively of modern and historical samples, with the latter being the most numerous of the five (n = 27). Among them, 19 modern individuals from both the south and north of the Alps belong to the most common haplotype. Conversely, only one modern Alpine ibex sample from the Swiss Eastern Alps shares its haplotype with three historical samples from Italy. All remaining historical samples in the haplogroups D and E carry unique haplotypes. As a consequence, average genetic distances for ancient ibex are about six times greater than that of historical ibex (0.6% compared with 0.1%), while those of modern ibex are just 0.04%, indicating poor genetic diversity in current populations (Supplementary Tables S4 and S5). The Alpine ibex specimen Ötzi_stomach shows a genetic distance ranging from 0.10% to 0.21% when compared to samples from the Luzern region, which cover a time span from 9,679 to 3,457 cal. BP. A similar genetic distance of 0.17% is observed when Ötzi_stomach is compared to the Na1 sample (5,445 - 4,963 cal. BP), which is geographically close to Luzern. Despite being territorially more similar to the Riparo Dalmeri samples, which cover a time span from 13,500 cal. BP to 11,500 cal. BP, Ötzi_stomach shows a mean genetic distance of 1.06% from them, which is identical to that observed between Riparo Dalmeri and the other ancient samples. Given isotopic and genomic evidence indicating that Ötzi lived most of his life south of the Alpine range[46], it is unlikely that the ibex remains found in his stomach originated north of the Alps. Nonetheless, the available data do not allow a reliable inference about the provenance of the animal, and further analyses would be required to explore the possibility of an ibex population turnover between 11,500 cal. BP and 5,000 BP.

Supplementary Table S4. Average genetic distances. Measures among the different *Capra ibex* groups are based on 16,716 bp homologous mitochondrial sequences.

Group	*Average genetic distance (%)*
Modern	0,041
Historic	0,1
All Ancient	0,653
Haplogroup A	0,134
Haplogroup B	0,126
Haplogroup C	0,091

Haplogroup D	0,044
Haplogroup E	0,014

Supplementary Table S5. Haplotype and nucleotide diversity in ibex populations

Population	*N. of samples*	*Haplotype number*	*Haplotype diversity*	*Segregation sites*	*Nucleotide diversity*
Modern	29	14	0,869	31	0,0003
Historic	7	6	0,952	48	0,0009
Ancient	6	6	1	66	0,001
Dalmeri	6	6	1	57	0,001

Supplementary Note 5: Genetic sex estimation

(Fontani, F., Iacovera, R.)

The genetic sex of the newly generated ibex data was first estimated by applying the so-called "Mittnik method"[50]. We generated mapping statistics from reads aligned to the whole genome sequence of the *Capra hircus*, Saanen breed (GCA_015443085) and calculated the normalized ratio of sequences mapping to each chromosome. We then compared the mean ratio of reads mapping the autosomes to the X chromosome (Rx) and generated sex estimates (Supplementary Table S6) by using an edited version of the script provided in[51].

Supplementary Table S6. Results of Rx calculation for sex assignment

ID	*Rx*	*95% CI*	*Sex assignment*
RD8158	0,734	0.687 – 0.782	Not assigned
RD8159	0,487	0.477 – 0.498	Male
RD8161	0,933	0.923 – 0.942	Female
RD8163	0,841	0.817 - 0.865	Female
RD8179	0,517	0.509 – 0.525	Male
RD8190	0,964	0.956 – 0.973	Female
RD8192	0,498	0.491 – 0.505	Male
RD8194	0,499	0.494 - 0.504	Male
RD8202	0,528	0.520 – 0.535	Male
RD8203	0,583	0.537 – 0.629	Possibly XY but not XX
RD8204	0,860	0.828 – 0.893	Female

We then used the "Skoglund method" to calculate the ratio of reads mapping to the Y-chromosome, manually setting the Ry male limit to 0.025 (Supplementary Table S7).

Supplementary Table S7. Results of Ry calculation for sex assignment

ID	*NchrY+ NchrX*	*NchrY*	*Ry*	*SE*	*95% CI*	*Assignment (–malelimit=0.025)*
RD8158	11751	313	0,026	0,001	0.023-0.029	Possibly XY but not XX
RD8159	93654	5082	0,054	0,0007	0.052-0.055	XY
RD8161	230575	458	0,002	0,0001	0.001-0.002	XX
RD8163	29556	342	0,011	0,0006	0.01-0.012	XX
RD8179	35097	1972	0,056	0,001	0.053-0.058	XY
RD8190	33498	79	0,002	0,0003	0.001-0.002	XX
RD8192	293567	17080	0,058	0,0004	0.057-0.059	XY
RD8194	378320	21465	0,056	0,0004	0.056-0.057	XY
RD8202	5282	370	0,07	0,003	0.063-0.076	XY
RD8203	3605	163	0,045	0,003	0.038-0.052	XY
RD8204	5674	77	0,013	0,001	0.010-0.016	Possibly XX but not XY

To further validate the genetic results, we replicated the methodology presented in[52] to calculate the Y-chromosomes to autosomes read depth ratio observed in each sample after mapping to the annotated genome of *C. hircus* Saanen. A standardized mean read depth was initially calculated by computing (read number/chromosome length)*100. Thus, we expected a ratio of standardized mean read depth of the autosomes to X chromosomes to be equal to 1 for female individuals. For males, an expected 2:1 ratio for read depth of autosomes compared to read depth on Y- and X-chromosomes was calculated (Supplementary Table S8).

Supplementary Table S8. Read depth statistics on Dalmeri ibex sequences mapped to sex and autosomal chromosomes

Id	*Read depth autosomes*	*Read depth X*	*Read depth Y*	*Inference*	*Ratio read depth autosomal – read depth X*	*Ratio read autosomal – read Y*
RD8158	0,012	0,008	0,003	Female	0,667	0,250
RD8159	0,128	0,062	0,052	Male	0,484	0,406
RD8161	0,174	0,161	0,004	Female	0,925	0,023
RD8163	0,025	0,02	0,003	Female	0,800	0,120
RD8179	0,045	0,023	0,02	Male	0,511	0,444
RD8190	0,02	0,023	0,001	Female	1,150	0,050
RD8192	0,363	0,194	0,177	Male	0,534	0,488
RD8194	0,467	0,25	0,223	Male	0,535	0,478
RD8202	0,006	0,003	0,003	Male	0,500	0,500
RD8203	0,005	0,002	0,001	Male	0,400	0,200
RD8204	0,004	0,003	0,001	Female	0,750	0,250

Supplementary references

1. Naudinot, N., Tomasso, A., Tozzi, C. & Peresani, M. Changes in mobility patterns as a factor of ^{14}C date density variation in the Late Epigravettian of Northern Italy and Southeastern France. *J. Archaeol. Sci.* **52**, 578–590 (2014).
2. Ravazzi, C., Peresani, M., Pini, R. & Vescovi, E. Il Tardoglaciale nelle Alpi italiane e in Pianura Padana. Evoluzione stratigrafica, storia della vegetazione e del popolamento antropico. *Alp. Mediterr. Quat.* **20**, 163–184 (2007).
3. Peresani, M., Duches, R., Miol, R., Romandini, M. & Ziggiotti, S. Small specialized hunting sites and their role in Epigravettian subsistence strategies: A case study in Northern Italy. In *Hunting Camps in Prehistory. Current Archaeological Approaches* (eds. Bon, F., Costamagno, S. & Valdeyron, N.) vol. 3 251–266 (2011).
4. Aimar, A. *et al.* Les Abris Villabruna dans la Vallée du Cismon. *Preistoria Alpina* **28**, 227–254 (1992).
5. Bertola, S. *et al.* L'Epigravettiano recente nell'area prealpina e alpina orientale. In *L'Italia tra 15.000 e 10.000 anni fa: Cosmopolitismo e regionalità nel tardoglaciale* (ed. Martini, F.) (Museo fiorentino di preistoria Paolo Graziosi, Firenze, 2007).
6. Finsinger, W., Tinner, W., Vanderknaap, W. & Ammann, B. The expansion of hazel (*Corylus avellana* L.) in the southern Alps: A key for understanding its early Holocene history in Europe? *Quat. Sci. Rev.* **25**, 612–631 (2006).
7. Vescovi, E. *et al.* Interactions between climate and vegetation during the Lateglacial period as recorded by lake and mire sediment archives in Northern Italy and Southern Switzerland. *Quat. Sci. Rev.* **26**, 1650–1669 (2007).
8. Tinner, W. & Vescovi, E. Ecologia e oscillazioni del limite degli alberi nelle Alpi dal Pleniglaciale al presente, Studi Trentini di Scienze Naturali. *Acta Geol.* **82**, 7–14 (2005).
9. Duches, R. *et al.* Trasformazione della mobilità epigravettiana in area alpina durante il Dryas recente: Il progetto YDESA. In *Archeologia delle Alpi* (ed. Nicolis, F.) (Provincia Autonoma, Soprintendenza Beni Culturali, Ufficio Beni Archeologici, Trento, 2014).

10. Mussi, M. & Peresani, M. Human settlement of Italy during the Younger Dryas. *Quat. Int.* **242**, 360–370 (2011).

11. Duches, R., Peresani, M. & Pasetti, P. Success of a flexible behavior. Considerations on the manufacture of Late Epigravettian lithic projectile implements according to experimental tests. *Archaeol. Anthropol. Sci.* **10**, 1617–1643 (2018).

12. Montoya, C. Apport de l'analyse technique à la compréhension de l'évolution des groupes humains épigravettiens d'Italie Nord Oriental: La production lithique de l'US 15a-65 du Riparo Dalmeri. *Preistoria Alpina* **43**, 191–208 (2008).

13. Montoya, C. & Peresani, M. Premiers éléments de diachronie dans l'Epigravettien récent des Préalpes de la Vénétie. In *D'un monde à l'autre. Les systèmes lithiques pendant le Tardiglaciaire autour de la Méditerranée nord-occidentale* (eds. Bracco, J. P. & Montoya, C.) 123–138 (2005).

14. Fasser, N., Visentin, D., Duches, R., Peresani, M. & Fontana, F. Lithic projectile technology in the western Late Epigravettian: The case study of north-eastern Italy. *Quat. Int.* **694**, 70–90 (2024).

15. Dalmeri, G. *et al.* Riparo Dalmeri: Le pietre dipinte dell'area rituale. *Preistoria Alpina* **45**, 67–117 (2011).

16. Fiore, I., Tagliacozzo, A. & Cassoli, P. F. Ibex exploitation at Dalmeri rockshelter (TN) and "specialized hunting" in the sites of the Eastern Alps during the Tardiglacial and the Early Holocene. *Preistoria Alpina* **34**, 173–183 (2001).

17. Villa, G. & Giacobini, G. Nuovi denti umani dai livelli epigravettiani di Riparo Dalmeri (TN). *Preistoria Alpina* **41**, 245–250 (2005).

18. Phoca-Cosmetatou, N. Specialisation & diversification: A tale of two subsistence strategies from Late Glacial Italy. *Before Farming* **3**, 1–29 (2009).

19. Fiore, I. & Tagliacozzo, A. Lo sfruttamento dello stambecco nel Tardiglaciale di Riparo Dalmeri (TN): Il livello 26c. In *Archaeozoological studies in honour of Alfredo Riedel* (Bolzano, 2006).

20. Dalmeri, G., Bassetti, M., Cusinato, A., Kompatscher, K. & Kompatscher Hrozny, M. The discovery of a painted anthropomorphic figure at Riparo Dalmeri and new insights into alpine Epigravettian art. *Preistoria Alpina* **41**, 163–169 (2005).

21. Fiore, I. & Tagliacozzo, A. Oltre lo stambecco: Gli altri mammiferi della struttura abitativa dell'US 26c a Riparo Dalmeri (Trento). *Preistoria Alpina* **43**, 209–236 (2008).
22. Lemorini, C. *et al.* L'analisi delle tracce d'uso e l'elaborazione spaziale: Il riconoscimento di un'area specializzata nel sito epigravettiano di Riparo Dalmeri, livelli 26b e 26c (Trento). *Preistoria Alpina* **41**, 171–197 (2006).
23. Dalmeri, G., Bassetti, M., Cusinato, A., Kompatscher, K. & Kompatscher Hrozny, M. Riparo Dalmeri: Un centro di sacralità in un sito epigravettiano di montagna (Trento). In *Preistoria dell'Italia settentrionale. Studi in ricordo di Bernardino Bagolini* 31–42 (Udine, 2006).
24. Dalmeri, G. *et al.* The ochre painted stones from the Riparo Dalmeri (Trento). Development of the research on the art and rituality of the Epigravettian site. *Preistoria Alpina* **44**, 95–119 (2009).
25. Bassetti, M. *et al.* Updating on the Final Palaeolithic-Mesolithic transition in Trentino (NE Italy). *Preistoria Alpina* **44**, 121–135 (2009).
26. Angelucci, D. E. *et al.* La successione esterna del Riparo Dalmeri (Trento, Italia). Prime informazioni geoarcheologiche. *Preistoria Alpina* **45**, 127–146 (2011).
27. Perrin, T. The time of the last hunters: Chronocultural aspects of Early Holocene societies in the Western Mediterranean. *Open Archaeol* **9**, 20220275 (2023).
28. Fontana, F., Flor, E. & Duches, R. Technological continuity and discontinuity in the Romagnano Loc III rock shelter (NE Italy) Mesolithic series. *Quat. Int.* **423**, 252–265 (2016).
29. Fiore, I. & Tagliacozzo, A. Riparo Cogola: Il contesto paleoecologico e lo sfruttamento delle risorse animali tra Epigravettiano e Mesolitico antico. *Preistoria Alpina* **40**, 159–186 (2005).
30. Cristiani, E. *et al.* Non-flaked stones used in the Mesolithic Eastern Alpine Region: A functional assessment from Romagnano Loc III and Pradestel sites. *J. Archaeol. Sci. Rep.* **37**, 102928 (2021).
31. Broglio, A. The discovery of the Mesolithic in the Adige Valley and the Dolomites (North-eastern Italy): A history of research. *Quat. Int.* **423**, 5–8 (2016).
32. Flor, E., Fontana, F. & Peresani, M. Contribution to the study of Sauveterrian technical systems. Technological analysis of the lithic industry from layers AF-AC1 of Romagnano Loc III rockshelter (Trento). *Preistoria Alpina* **45**, 193–219 (2011).

33. Boscato, P., Broglio, A., Cattani, L., Perini, R. & Sala, B. Romagnano III. *Preistoria Alpina* **28**, 275–284 (1992).

34. Broglio, A. & Kozlowski, S. K. Tipologia ed evoluzione delle industrie mesolitiche di Romagnano III. *Preistoria Alpina* **19**, 93–148 (1983).

35. Broglio, A. Risultati preliminari delle ricerche sui complessi epipaleolitici della Valle dell'Adige. *Preistoria Alpina* **7**, 135–241 (1971).

36. Stuiver, M. & Reimer, P. J. Extended ^{14}C data base and revised CALIB 3.0 ^{14}C age calibration program. *Radiocarbon* **35**, 215–230 (1993).

37. Fiore, I., Gala, M., Dalmeri, G., Duches, R. & Tagliacozzo, A. Bird exploitation from the epigravettian site of Riparo Dalmeri (Trento, Italy). *Quat. Int.* **626–627**, 33–42 (2022).

38. Tagliacozzo, A. & Fiore, I. Hunting strategies in a mountain environment during the Late Glacial in north eastern Italy. *Preistoria Alpina* **44**, 79–93 (2009).

39. Fiore, I. & Tagliacozzo, A. Lo sfruttamento delle risorse animali nei siti di altura e di fondovalle nel Tardiglaciale dell'Italia nord-orientale. In *Preistoria Alpina* (eds. Malerba, G. & Visintini, P.) 97–109 (2005).

40. Fiore, I. & Tagliacozzo, A. L'analisi dei resti faunistici: Il contesto paleoecologico e l'economia del sito. In *Pitture paleolitiche nelle prealpi venete, Grotta di Fumane e Riparo Dalmeri* (eds. Broglio, A. & Dalmeri, G.) (2005).

41. Tagliacozzo, A. & Fiore, I. Chasse specialisee dans une site de montagne: L'exemple de l'Abri Dalmeri (Trento, Italie). In *La gestion demografhique des animaux a travers le temps* 69–76 (Ibex Journal of Mountain Ecology 5, Anthropozoologica 31, 2000).

42. Cassoli, P. F., Dalmeri, G., Fiore, I. & Tagliacozzo, A. La chasse dans un gisement Epigravettien de montagne: Riparo Dalmeri (Trento, Italie). In *L'Europe des derniers chasseurs. Epipaleolithique et Mesolithique: Peuplement et paleonvironnement de l'Epipaleolithique et du Mesolithique* (eds. Thevenin, A. & Bintz, P.) 459–464 (Editions du CTHS, Paris, 1999).

43. Albertini, D. & Tagliacozzo, A. Fresh water fishing in Italy during the Late Glacial period: the example of Riparo Dalmeri. In *Petits animaux et sociétés humaines. Du complément alimentaire aux ressources utilitaires* (eds. Brugal, J. P. & Desse, J.) 131–136 (Ed. APDCA, Antibes, 2004).

44. Curci, A. & Tagliacozzo, A. Determinazione dell'età di morte e della stagione di cattura attraverso lo studio dei livelli di accrescimento di cemento e dentina nei denti di mammiferi: l'esempio di Riparo Dalmeri (TN). In *Atti del 2° Convegno Naizonale di Archeozoologia (Asti, 1997)* 23–30 (ABACO Edizioni, Forlì, 2000).
45. Larkin, M. A. *et al.* Clustal W and Clustal X version 2.0. *Bioinformatics* **23**, 2947–2948 (2007).
46. Keller, A. *et al. New insights into the Tyrolean Iceman's origin and phenotype as inferred by whole-genome sequencing. Nat. Commun.* **3**, 698 (2012).
47. Peng, J. *et al.* Comparison of Database Searching Programs for the Analysis of Single-Cell Proteomics Data. *J. Proteome Res.* **22**, 1298–1308 (2023).
48. Mackie, M. *et al.* Palaeoproteomic profiling of conservation layers on a 14th century Italian wall painting. *Angew. Chem. Int. Ed.* **57**, 7369–7374 (2018).
49. Cappellini, E. *et al.* Early Pleistocene enamel proteome from Dmanisi resolves *Stephanorhinus* phylogeny. *Nature* **574**, 103–107 (2019).
50. Mittnik, A., Wang, C.-C., Svoboda, J. & Krause, J. A molecular approach to the sexing of the triple burial at the Upper Paleolithic site of Dolní Věstonice. *PLoS ONE* **11**, e0163019 (2016).
51. De Flamingh, A., Coutu, A., Roca, A. L. & Malhi, R. S. Accurate sex identification of ancient elephant and other animal remains using low-coverage DNA shotgun sequencing data. *G3 (Bethesda)* **10**, 1427–1432 (2020).
52. Denoyelle, L. *et al.* VarGoats project: A dataset of 1159 whole-genome sequences to dissect Capra hircus global diversity. *Genet. Sel. Evol.* **53**, 86 (2021).
